# Primary biliary cholangitis has causal effects on systemic rheumatic diseases: a Mendelian randomization study

**DOI:** 10.1186/s12876-024-03319-3

**Published:** 2024-08-29

**Authors:** Hai-Ping Zhang, Zhe Zhou, Ke Chen, Li-Fen Xiong, Jun Wu, Lei Jin

**Affiliations:** 1 Department of Gastroenterology, Hubei NO. 3 People’s Hospital of Jianghan University, Wuhan, 430000 China; 2 Department of Radiology, The Affiliated Hospital of Wuhan Sports University, Wuhan, 430079 China

**Keywords:** Mendelian randomization, Primary biliary cholangitis, Systemic rheumatic diseases

## Abstract

**Background:**

An association has been observed between primary biliary cholangitis (PBC) and systemic rheumatic diseases (SRDs) in observational studies, however the exact causal link remains unclear. We aimed to evaluate the causal effects of PBC on SRDs through Mendelian randomization (MR) analysis.

**Methods:**

The genome-wide association study (GWAS) summary data were obtained from MRC IEU OpenGWAS and FinnGen databases. Independent genetic variants for PBC were selected as instrumental variables. Inverse variance weighted was used as the main approach to evaluate the causal effects of PBC on Sjögren syndrome (SS), rheumatoid arthritis (RA), systemic lupus erythematosus (SLE), systemic sclerosis (SSc), mixed connective tissue disease (MCTD) and polymyositis (PM). Horizontal pleiotropy and heterogeneity were measured by MR‒Egger intercept test and Cochran’s *Q* value, respectively.

**Results:**

PBC had causal effects on SS (*OR* = 1.177, *P* = 8.02e-09), RA (*OR* = 1.071, *P* = 9.80e-04), SLE (*OR* = 1.447, *P* = 1.04e-09), SSc (*OR* = 1.399, *P* = 2.52e-04), MCTD (*OR* = 1.306, *P* = 4.92e-14), and PM (*OR* = 1.416, *P* = 1.16e-04). Based on the MR‒Egger intercept tests, horizontal pleiotropy was absent (all *P* values > 0.05). The robustness of our results was further enhanced by the leave-one-out method.

**Conclusions:**

Our research has provided new insights into PBC and SRDs, indicating casual effects on various SRDs.

**Supplementary Information:**

The online version contains supplementary material available at 10.1186/s12876-024-03319-3.

## Introduction

Primary biliary cholangitis (PBC) is an autoimmune hepatobiliary disease characterized by destroyed interlobular bile ducts, leading to liver cirrhosis and an increased need for liver transplantation [1]. PBC is predominantly found in females, and its prevalence is increasing [2]. The estimated worldwide incidence and prevalence of PBC are 1.76 and 14.6 cases per 100 000 individuals, respectively [3]. Currently, there are limited treatments available for PBC and UDCA remains the primary first-line therapy, which may moderate disease progression and prolong transplant-free survival in PBC patients. Although extensive studies have been conducted on the immunological processes that cause liver injury [4, 5], the mechanism of PBC is still poorly understood. Therefore, it is essential to clarify the fundamental pathological mechanisms to develop more practical therapeutic strategies for PBC, as well as for extrahepatic complications.

Patients with PBC usually have various extrahepatic manifestations, particularly systemic rheumatic diseases (SRDs) [6, 7]. SRDs are chronic, inflammatory, autoimmune disorders with the presence of autoantibodies which may damage various systems. In a recent retrospective cohort of 1554 PBC individuals, the prevalence of SRDs was 17% [8]. Furthermore, patients with PBC frequently have autoantibodies associated with SRDs [9]. To date, coexisting PBC and SRDs have been reported in many observational studies [6–10], and the association between PBC and SRDs has received considerable scientific attention. The co-occurrence of PBC and SRDs suggests the possibility of common pathogenic mechanisms [9, 10]. However, the causal associations between PBC and SRDs are still unclear and further efforts are urgently needed to assess the causality between these disorders and develop prevention strategies.

It is challenging to draw valid conclusions about the causal associations between PBC and SRDs in traditional observational studies due to unadjusted confounders variables and reverse causality. Using genetic variation as instrumental variables (IVs) in Mendelian randomization (MR) analysis has been proven to be an effective technique in epidemiologic research for evaluating the exposure’s causal effects on outcomes [11]. At the time of gametogenesis, the genetic variations are assigned in a random manner, potentially reducing the impact of confounding factors. MR is particularly significant in epidemiological research, as it strengthens the ability to establish causality by minimizing confounder effects, providing more robust and reliable insights into the relationships between diseases. This investigation aimed to explore the causal link between PBC and SRDs through two-sample MR analysis, indicating positive causal effects of PBC on SRDs.

## Materials and methods

### Data sources

To eliminate genetic bias arising due to ethnic differences, the MR analysis was performed only in the European population. The genome-wide association study (GWAS) summary data for PBC [12] were obtained from the MRC IEU OpenGWAS project (https://gwas.mrcieu.ac.uk/). All PBC cases fulfilled the American Association for the Study of Liver Disease criteria for PBC. The GWAS summary data for SRDs (Sjögren syndrome [SS], rheumatoid arthritis [RA], systemic lupus erythematosus [SLE], systemic sclerosis [SSc], mixed connective tissue disease [MCTD] and polymyositis [PM]) were collected from the FinnGen consortium (R9 version) [13]. SRDs were classified using International Classification of Diseases (ICD) codes. The detailed information of GWAS data was shown in Table 1.

**Table 1:** Included GWAS summary data

Trait	Case	Control	Diagnosis criteria or ICD codes
PBC	8201	16,489	All cases fulfilled the American Association for the Study of Liver disease criteria for PBC
RA	12,555	240,862	ICD-10: M05, M06; ICD-9: 7140 A, 7140B, 7241, 7142; ICD-8: 7121,7172,7123
SS	2495	365,533	ICD-10: M35.0; ICD-9: 7102; ICD-8: 73,490
SLE	1023	281,127	ICD-10: M32, M32.8, M32.9; ICD-9: 7100; ICD-8: 7431
SSc	194	376,670	ICD-10: M34
MCTD	1849	375,428	ICD-10: M35.9; ICD-9: 7109; ICD-8: 73,499
PM	225	365,533	ICD-10: M33.2; ICD-9: 7104; ICD-8: 7161

### Study design

The MR selected single-nucleotide polymorphisms (SNPs) as IVs to assess the causal associations between PBC and SRDs. Our study fulfilled the following three main hypotheses: (1) IVs had a strong correlation with exposure; (2) IVs had no associations with any confounders; and (3) IVs could only affect outcomes by associating with exposure, not directly affecting outcomes. Therefore, the included SNPs were of genome-wide significance (*P* < 5 × 10e-8) without linkage disequilibrium (*r*^2^ < 0.001). The F-statistic was calculated according to a previously published MR study [14]. To avoid the bias of weak IVs, SNPs with an F-statistic below 10 were eliminated. We also excluded SNPs with palindromic alleles to avoid strand ambiguity and potential misinterpretation of the results. PhenoScanner database was used to screen and exclude SNPs that are related to confounders and outcomes.

### Statistical analysis

To investigate causal effects, the inverse variance weighted (IVW) approach was performed as the main analysis using the TwoSampleMR package in R software (version 4.3.1). IVW combined the Wald estimates of causality for each IV to obtain overall estimates of the effect of exposure on outcome. Two complementary approaches (MR‒Egger and weighted median [WME]) were used to validate the IVW results. The MR‒Egger regression method carried out a weighted linear regression to produce a consistent estimate of the causal effect, independent of the validity of the SNPs. WME can provide reliable estimates when more than 50% of the weight is derived from valid SNPs. Outliers were identified by the MR-PRESSO package based on the *p* value of the outlier test, and subsequent causal estimates were calculated after outlier removal.

To ensure that the SNPs were consistent with the basic assumptions of the MR analysis, the MR‒Egger intercept test was used to assess the possible pleiotropic effects of the SNPs. Cochran’s *Q* value was used to quantify heterogeneity, and the presence of heterogeneity was determined if the *P* value was < 0.05. The IVW fixed effects method was used if the *P* value of the Cochran’s *Q* test was < 0.05, otherwise the random effects model was used. In addition, the robustness of the MR results was validated using the leave-one-out approach.

## Results

After a series of strict quality control procedures, included SNPs for assessing the causal effects of PBC on SRDs were provided in the Supplemental Tables. The F-statistic values of included SNPs were more than 10, indicating that the IVs have sufficient power.

**Fig. 1:**
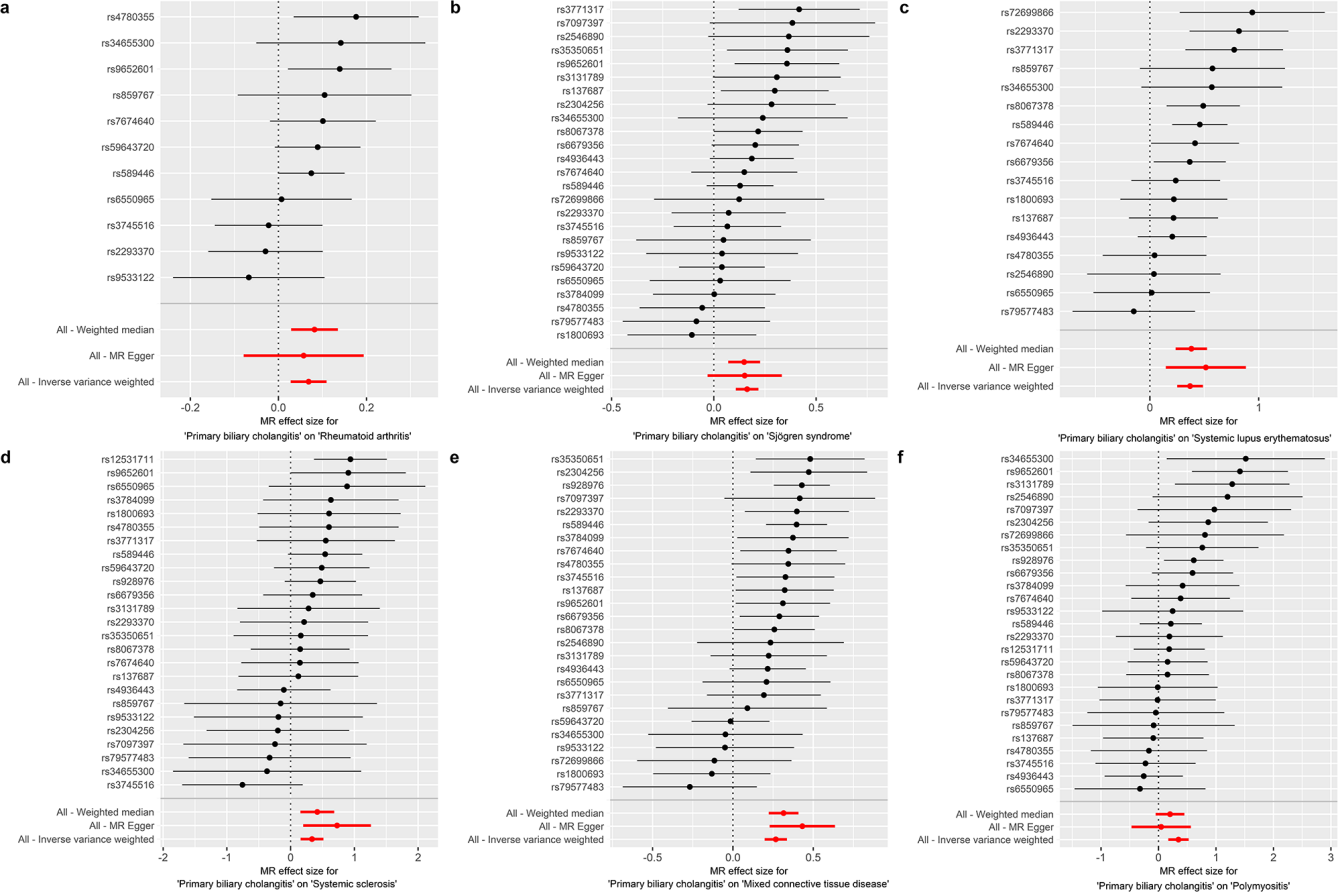
Causal effects of PBC on various SRDs. Causal effects of PBC on RA (**a**), SS (**b**), SLE (**c**), SSc (**d**), MCTD (**e**) and PM (**f**)

The causal relationships between PBC and SRDs were summarized in Table 2; Fig. 1. The IVW analysis showed that genetically predicted PBC had causal effects on RA (OR = 1.071, *P* = 9.80e-04), SS (OR = 1.177, *P* = 8.02e-09), SLE (OR = 1.447, *P* = 1.04e-09), SSc (OR = 1.399, *P* = 2.52e-04), MCTD (OR = 1.306, *P* = 4.92e-14) and PM (OR = 1.416, *P* = 1.16e-04). Consistent results were obtained by the WME method, showing that PBC was positively associated with RA (OR = 1.085, *P* = 2.88e-03), SS (OR = 1.160, *P* = 3.19e-04), SLE (OR = 1.464, *P* = 3.06e-07), SSc (OR = 1.519, *P* = 1.34e-03) and MTCD (OR = 1.371, *P* = 1.15e-12). MR‒Egger also verified the causal effects of PBC on SLE (OR = 1.672, *P* = 1.52e-02), SSc (OR = 2.072, *P* = 1.31e-02) and MTCD (OR = 1.540, *P* = 3.51e-04), and showed the same direction of causal effects on RA (OR = 1.059, *P* = 0.431), SS (OR = 1.163, *P* = 0.117) and PM (OR = 1.048, *P* = 0.860).

**Fig. 2:**
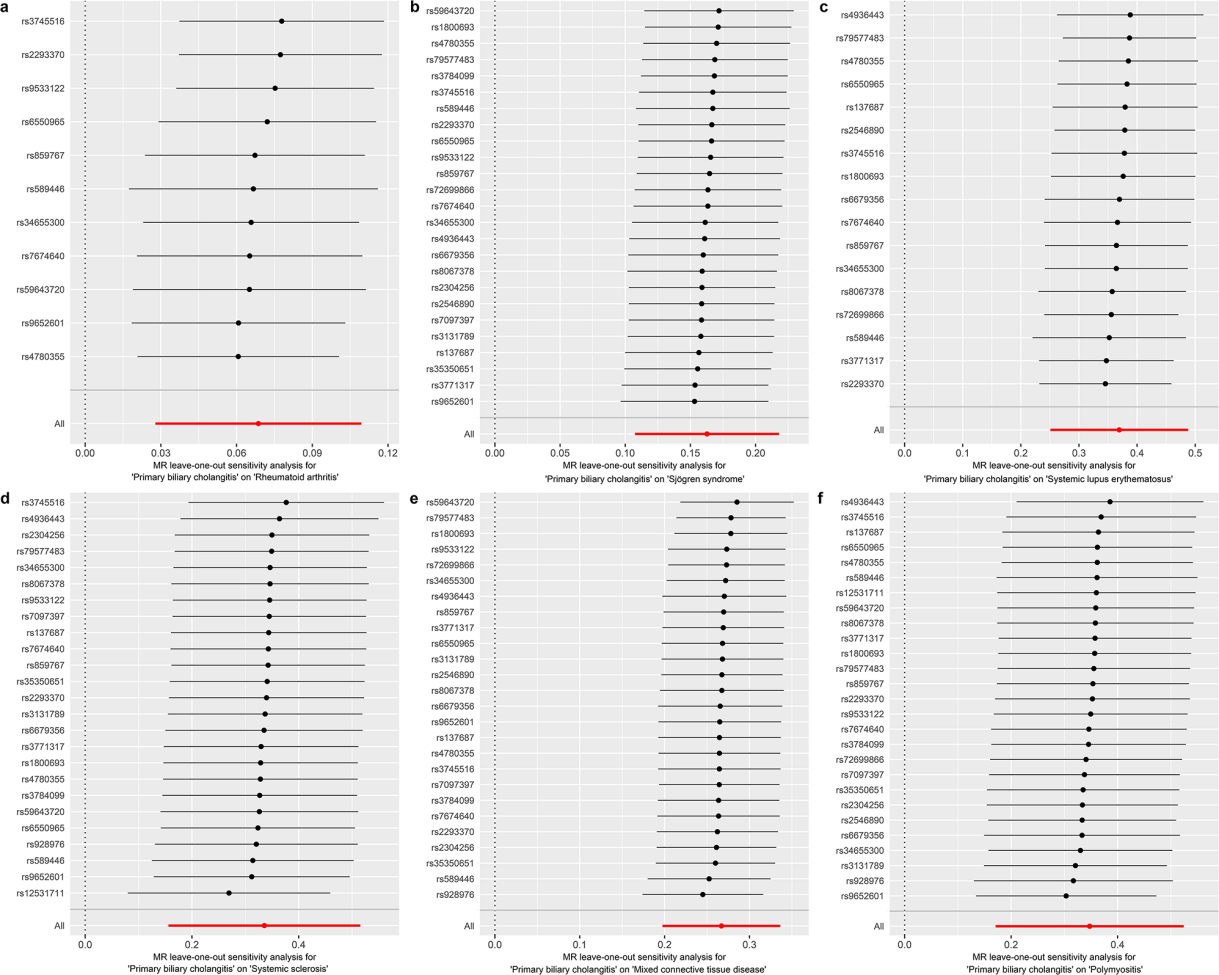
“Leave-one-out” sensitivity analysis. Sensitivity analysis for causal effects of PBC on RA (**a**), SS (**b**), SLE (**c**), SSc (**d**), MCTD (**e**) and PM (**f**)

The included SNPs did not show any heterogeneity (Table 2). According to the MR‒Egger intercept tests, there was no horizontal pleiotropy in the present MR study (Table 2). Furthermore, the robustness of the MR analysis results was confirmed by the “leave-one-out” method (Fig. 2).

**Table 2:** **Causal effects of PBC on various SRDs**.

Outcomes	Methods	OR(95%CI)	*P* value	Cochran’s Q (*P* value)	MR‒Egger intercept (*P* value)
RA	MR‒Egger	1.059(0.924–1.214)	4.31E-01	12.009(0.212)	0.003(0.868)
	IVW	1.071(1.028–1.116)	9.80E-04	12.048(0.282)	
	WME	1.085(1.028–1.146)	2.88E-03		
SS	MR‒Egger	1.163(0.970–1.395)	1.17E-01	23.347(0.441)	0.003(0.894)
	IVW	1.177(1.113–1.244)	8.02E-09	23.366(0.498)	
	WME	1.160(1.070–1.257)	3.19E-04		
SLE	MR‒Egger	1.672(1.158–2.416)	1.52E-02	20.702(0.147)	-0.036(0.427)
	IVW	1.447(1.285–1.629)	1.04E-09	21.622(0.156)	
	WME	1.464(1.265–1.694)	3.06E-07		
SSc	MR‒Egger	2.072(1.219–3.525)	1.31E-02	17.701(0.773)	-0.103(0.137)
	IVW	1.399(1.169–1.674)	2.53E-04	20.078(0.692)	
	WME	1.519(1.177–1.962)	1.34E-03		
MCTD	MR‒Egger	1.540(1.257–1.888)	3.51E-04	29.782(0.192)	-0.041(0.105)
	IVW	1.306(1.218-1.400)	4.92E-14	33.302(0.124)	
	WME	1.371(1.257–1.495)	1.15E-12		
PM	MR‒Egger	1.048(0.626–1.756)	8.60E-01	27.821(0.316)	0.077(0.236)
	IVW	1.416(1.186–1.689)	1.16E-04	29.461(0.291)	
	WME	1.223(0.956–1.564)	1.09E-01		

## Discussion

Our research focused on the causal effect of PBC on SRDs, an issue previously unresolved in observational epidemiological studies. To the best of our knowledge, this is the first MR study to systemically assess the causal links between PBC and various SRDs. Although the potential mechanisms underlying PBC and SRDs were not fully understood, our study provided compelling evidence for the causal relationship between them. Heterogeneity and horizontal pleiotropy were absent in the present MR study. The reliability of our MR findings was demonstrated by sensitivity analysis. Our results indicate potential overlap in the pathophysiological mechanisms underlying these autoimmune diseases.

A close association has been observed between PBC and SRDs in previous observational studies. The prevalence of SRDs in PBC varied greatly in different cohorts. The reported prevalences of SS, RA, SLE, SSc, MCTD and PM in PBC are 3.5–73%, 1.8–13%, 1.3–3.7%, 1.4–12.3%, 0.6–3.1%, and 0.6% [6, 7], respectively. Serum autoantibodies associated with SRDs are often detected in PBC [9, 15], including rheumatoid factor, anti-CCP, anti-dsDNA, anti-Ro/SSA, etc. Antinuclear antibodies (ANAs) exhibiting the “multiple nuclear dots” or “rim-like immunofluorescence” patterns showed high specificity for PBC and these ANAs were also detected in SRDs [16]. Though the previous studies have reported the co-existence of PBC and SRDs, our study provides stronger evidence for a causal relationship. Our study employed MR approach to evaluate the causal associations between PBC and SRDs, showing positive causal effects of PBC on SRDs.

The liver, as a major lymphoid organ, plays a crucial role in immune surveillance and regulation through its abundant lymphocytes and Kupffer cells, which may explain the observed associations between PBC and SRDs. The development of extrahepatic autoimmune disorders involves the interactions of both natural and adaptive immune responses that target cholangiocytes and various extrahepatic tissues [17]. It has been demonstrated that the L-12/IL-23-mediated Th1/Th17 signaling pathway plays important role in the development of PBC. A shift in the balance of Th1 to Th17 cells has been observed and is thought to contribute to the unfavorable disease progress [18]. Th1 and Th17 cells in adaptive immune responses also play crucial roles in SRDs [19–21]. For example, elevated serum levels of Th1 related cytokines, such as IFN-γ, IL12 and IL18, are detected in SLE patients, and high levels of cytokines were positively associated with disease severity [22]. Th17 cells in SLE and SS are associated with increased production of IL-17, which promotes inflammation and autoantibody production [23, 24], further driving the autoimmune process. Besides, smoking is considered as a common risk factor for PBC and SRDs [25–28]. Smoking has been demonstrated to impair immune function through various mechanisms, including the induction of the inflammatory response, immune suppression, alteration of cytokine balances, which contribute to the development of various autoimmune diseases [29, 30]. Given the findings in the present MR analysis, it is imperative to implement effective screening strategies for the early detection of SRDs in PBC patients, particularly in those with a smoking habit.

There is growing evidence to suggest that there is a strong link between gut dysbiosis and autoimmunity [31, 32]. Perturbations in the gut microbiota could lead to the production of pro-inflammatory cytokines and an increase in Th17 cells, even in extraintestinal tissues. Patients with autoimmune diseases have different gut microbiota compositions compared to healthy subjects [33, 34]. Decreased bacterial diversity was observed in PBC cases and gut dysbiosis is associated with clinical prognosis [35, 36]. Alterations in the microbiota composition, such as Faecalibacterium and Lactobacillus, were found in PBC, RA, SLE, SS and SSc [32–34]. In addition, changes in the gut microbiota are correlated with the severity of SRDs and are being developed as a novel diagnostic method for several SRDs. To date, no research has yet been done on the microbiome of MCTD and polymyositis. Animal and clinical studies have shown promising results for gut microbiota-based therapy, supporting the hypothesis that changes in gut microbiota can affect autoimmune responses and disease outcomes [37]. However, the gut microbiota composition is frequently impacted by dietary and ecological factors, and further research with better matching controls is needed. In addition, potential mechanisms of microbiota urgently need to be urgently verified to explore the interaction between PBC and SRDs.

Considerable efforts have been made to investigate the genetic inheritance of PBC and SRDs [38, 39], highlighting the vital importance of genetic background between these diseases. Previous GWAS showed that PBC and SRDs shared some common genes involved in the IL12-mediated signaling pathway, including STAT4 and IRF5 [38, 39]. STAT4 and IRF5 are key transcription factors activated by IL-12 signaling, which promotes Th1 differentiation and IFN-γ production [40, 41]. These molecules are crucial in driving the inflammatory response and are implicated in the pathogenesis of both PBC and SRDs. These findings may provide an explanation for why individuals with common genes are more susceptible to concurrent SRDs in PBC. Osteopontin (OPN) is a versatile cytokine and adhesion molecule, acting as a unique regulator of both innate and adaptive immune responses [42]. OPN has a vital role in recruiting mononuclear cells to epithelioid granulomas and participating in bile duct injury through B-cell differentiation and plasma cell expansion in PBC [43]. OPN expression is upregulated in SS, RA, SLE and SSc, and OPN overexpression has been shown to be correlated with disease severity [42, 44–46]. For example, OPN overexpression has been associated with a predisposition to SLE and poor prognosis since OPN promotes the T follicular helper cells and enhances anti-nuclear antibody production [44]. Further comprehension of the genetic heredity of these disorders will be beneficial for clinical pharmacotherapy.

Although large samples of GWAS summary data were used for MR analysis, several limitations of this research should be recognized. First, the MR results in the present study were obtained from the European population. The causal association between PBC and SRDs remains inconclusive in other ethnic groups. Second, both PBC and SRDs are female-predominant. Due to the currently limited GWAS data, stratification of the genetic causal effects between PBC and SRDs by gender could not be explored. Third, only six SRDs were analyzed in this study. While our study focused on six SRDs, the possibility of causal relationships between PBC and other SRDs could not be excluded. Additionally, due to the lack of effective IVs for most SRDs in reverse MR analyses, causal effects of SRDs on PBC were not assessed. Finally, MR analysis only provides the causal association between PBC and SRDs, without explaining the biological process behind the connection. Hence, further experimental efforts are needed to confirm these findings.

## Conclusions

The MR results show positive causal effects of PBC on SRDs, which holds significant clinical guidance for clinicians in daily medical practice. It may be advisable to regularly screen SRDs for patients with PBC, which may facilitate earlier diagnosis and timely management. Additionally, elucidating the shared genetic and biological mechanisms between these conditions can pave the way for novel therapeutic targets and personalized treatment approaches. In the future, multidisciplinary cooperation is essential for the satisfactory management of these patients.

### Electronic supplementary material

Below is the link to the electronic supplementary material.

Supplementary Material 1

## Data Availability

All GWAS data are publicly available in the MRC IEU OpenGWAS database (https://gwas.mrcieu.ac.uk/) and FinnGen database (http://www.finngen.fi).
